# Effect of Early Postoperative Gait Parameters After Total Hip Arthroplasty on Forgotten Joint Score-12 at 2-Year Follow-Up

**DOI:** 10.3390/geriatrics10010007

**Published:** 2025-01-06

**Authors:** Kazuya Okazawa, Satoshi Hamai, Tsutomu Fujita, Shinya Kawahara, Daisuke Hara, Yasuharu Nakashima, Hiroshi Katoh

**Affiliations:** 1 Department of Rehabilitation, Kyushu University Hospital, 3-1-1 Maidashi, Higashi-ku, Fukuoka 812-8582, Japan; fujita.tsutomu.271@m.kyushu-u.ac.jp; 2 Graduate School of Health Sciences, Yamagata Prefectural University of Health Sciences, 260 Kamiyanagi, Yamagata 990-2212, Japan; hikato@yachts.ac.jp; 3 Department of Orthopedic Surgery, Graduate School of Medical Sciences, Kyushu University, 3-1-1 Maidashi, Higashi-ku, Fukuoka 812-8582, Japan; shamai0220@gmail.com (S.H.); kawahara.shinya.310@m.kyushu-u.ac.jp (S.K.); hara.daisuke.605@m.kyushu-u.ac.jp (D.H.); nakashima.yasuharu.453@m.kyushu-u.ac.jp (Y.N.)

**Keywords:** total hip arthroplasty, forgotten joint score-12, gait parameters

## Abstract

**Purpose:** This study aimed to elucidate the relationship between early postoperative gait parameters after total hip arthroplasty (THA) and the Forgotten Joint Score-12 (FJS-12) score at the 2-year follow-up after surgery. In addition, the cutoff gait parameter values for predicting discomfort using specific FJS-12 subitems were evaluated. **Methods:** Among 313 patients who underwent THA between April and December 2019, 44 (14.0%) who responded to the FJS-12 questionnaire at 2 years postoperatively were included in this study. Gait parameters, including walking speed, stride length, and their coefficients of variation (CVs), were measured at 13.8 ± 3.6 (mean ± standard deviation) days postoperatively. The FJS-12 was used to evaluate patients at 2 years after surgery. The correlation between the FJS-12 score and gait parameters was analyzed using Spearman’s rank correlation coefficient. To determine the significant predictors of the FJS-12 score, multiple regression analysis was performed after adjusting for age as a covariate. Furthermore, receiver operating characteristic curves were used to determine the cutoff gait parameter values for predicting discomfort using specific FJS-12 subitems. **Results:** The FJS-12 score was significantly positively correlated with walking speed (r_s_ = 0.38, *p* < 0.05) and stride length (r_s_ = 0.51, *p* < 0.01). Meanwhile, the FJS-12 score was significantly negatively correlated with the CVs of walking speed (r_s_ = −0.34, *p* < 0.05) and stride length (r_s_ = −0.35, *p* < 0.05). Based on multiple regression analysis, stride length was a significant predictor of discomfort assessed using the FJS-12 score (β = 0.48, *p* < 0.01). According to the receiver operating characteristic curves, the cutoff stride length values for predicting discomfort using the FJS-12 subitems 9, 10, 11, and 12 showed moderate accuracy (area under the curve > 0.7). **Conclusions:** Improved walking ability of patients who underwent THA through early rehabilitation is linked to joint discomfort and patient satisfaction in daily life 2 years postoperatively.

## 1. Purpose

Hip osteoarthritis (HOA) commonly affects middle-aged and older adults and causes severe pain, reduced range of motion, and muscle weakness [1,2]. Total hip arthroplasty (THA) is commonly performed as a surgical treatment of HOA [3]. Recently, the Forgotten Joint Score-12 (FJS-12), a patient-reported outcome measure (PROM), has been increasingly used as a postoperative evaluation method for THA [4,5,6,7,8,9]. The FJS-12 quantifies postoperative patient satisfaction, with higher scores indicating a greater degree of forgetting the operated joint during daily activities and movement [10].

Previous studies have shown that subjective leg length discrepancy at 1 year after THA and decreased quality of the iliopsoas muscle on computed tomography are associated with lower FJS-12 scores and functional impairment [11,12]. Rehabilitation after THA is important for improving gait ability and enhancing the quality of life of patients. Walking speed is a key parameter in gait ability assessment. Hence, it is an important predictor of outcomes such as length of hospital stay, gait patterns, and fall risk [13,14].

Previous research has explored the relationship between biomechanical parameters (such as decreased limb joint moments) and PROMs such as the Japanese Orthopedic Association Hip Disease Evaluation Questionnaire in patients who underwent THA [15]. However, no studies have examined the relationship between the FJS-12 score and kinematic parameters (such as time and distance) during walking.

Therefore, this study aimed to examine the relationship between early postoperative gait parameters (such as time and distance) and the FJS-12 score in patients who underwent THA. Moreover, the cutoff gait parameter values for predicting discomfort using specific FJS-12 items at 2 years after THA were evaluated.

## 2. Methods

### 2.1. Study Design and Participants

**Figure 1:**
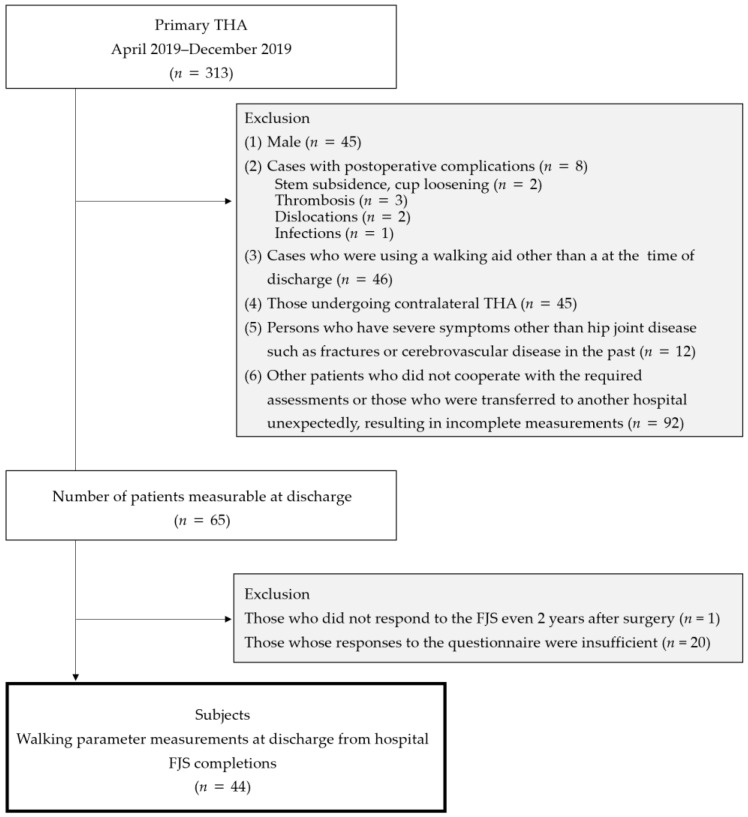
PRISMA flow diagram for study selection.

This was a single-center retrospective observational study. In total, 313 patients underwent THA at our hospital between April and December 2019. All THAs were performed using a posterolateral approach, with postoperative rehabilitation conducted according to a standardized protocol. The exclusion criteria were as follows: (1) male sex, (2) postoperative complications, (3) use of walking aids other than a cane at discharge, (4) history of contralateral THA, (5) severe conditions other than hip disease (e.g., fractures or cerebrovascular disease), and (6) patients who did not cooperate with assessments or were transferred to another hospital, resulting in incomplete measurements. In total, 65 (20.7%) patients who provided consent and underwent measurements at discharge were included in this study. The FJS-12 questionnaire was mailed to the patients at 2 years postoperatively, and 44 (14.0%) patients who provided responses were included in the final analysis (Figure 1).

### 2.2. Data Collection

Patient demographic data, including age at the time of surgery, height, weight, body mass index, and surgical information, were collected from the electronic medical records (Table 1). Gait parameters, including walking speed, stride length, and their coefficients of variation (CVs), were measured on the day before discharge, i.e., at 13.8 ± 3.6 (mean ± standard deviation) days postoperatively.

The primary outcome measure was the FJS-12 score at 2 years postoperatively. The participants’ responses to the FJS questionnaire were collected and analyzed to determine the relationship between early postoperative gait parameters and the FJS-12 score.

### 2.3. Measurement of Gait Parameters

The gait parameters included walking speed (cm/s), stride length (cm), walking speed CV (%), and stride length CV (%). Higher CV values indicated greater gait variability, which is considered an indicator of gait stability [16,17].

Measurements were obtained using Walkway MW-1000 (Anima Co., Ltd., Tokyo, Japan), a sheet-type foot pressure measurement device (width × length, 60 cm × 7.2 m). Based on previous studies [18], a 10 m walkway was set up, and each patient was instructed to walk naturally along it four times. The device was positioned in the middle of the walkway, where it recorded the footfalls. Each walk provided an average of 3.9 ± 0.4 gait cycles.

### 2.4. FJS-12

The FJS-12 is a 12-item questionnaire used to evaluate awareness of the joint in daily life after arthroplasty [19] (Table 2). Each item assesses the frequency of joint awareness on a 5-point scale: never—0 points; almost never—1 point; seldom—2 points; sometimes—3 points; and mostly—4 points. The total score is converted based on a 100-point scale, with higher scores indicating better outcomes.

Based on previous research, the scores in this study were categorized into two groups: no discomfort (0–2) and discomfort (3–4). The FJS-12 score is considered a highly discriminative outcome measure [20] and has a high internal validity for THA and total knee arthroplasty [21].

The FJS-12 scores are assigned as follows: a score of 0 points for “never”, 1 for “almost never”, 2 for “seldom”, 3 for “sometimes”, and 4 for “mostly”. To obtain the final score, the average score of the 12 items is multiplied by 25 and then subtracted from 100, with 0 indicating the minimum score and 100 indicating the maximum score. A higher total score reflects limited awareness of the artificial joint.

### 2.5. Statistical Analysis

Data normality was assessed using the Shapiro–Wilk test. The correlation between the FJS-12 score and each gait parameter was evaluated using Spearman’s rank correlation coefficient (r_s_). Next, a stepwise multiple regression analysis was conducted to examine the relationship between gait parameters and FJS. This analysis used gait parameters that were significantly correlated with the FJS-12 score, an independent variable, and age, a covariate for adjusting potential confounding effects. Multicollinearity among variables was assessed using the variance inflation factor. Furthermore, to identify the cutoff values for predicting discomfort using the FJS-12 items, the presence or absence of discomfort in each item was used as a dependent variable. Receiver operating characteristic (ROC) curves were employed, with an area under the curve (AUC) used to determine model fit. The cutoff value was set at the point where the Youden index was maximized, and the sensitivity and specificity were calculated. The accuracy of the AUC was classified as high (>0.9), moderate (0.9–0.7), or low (0.7–0.5) [22]. To assess the validity of the findings, post hoc power analysis of the correlation and multiple regression analyses was conducted using G*POWER 3.1.9.7 and the Cohen’s method [23]. Statistical analyses were performed using the JMP software (version 17.0, SAS Institute Inc., Cary, NC, USA), and the significance level was set at 5%.

## 3. Results

### 3.1. FJS-12 Score and Gait Parameters

The mean FJS-12 score of the participants was 62.3 ± 29.7. The gait parameters measured were as follows: walking speed—80.9 ± 21.1 cm/s; stride length—96.5 ± 17.6 cm; walking speed CV—7.6% ± 4.6%; and stride length CV—6.4% ± 5.1%. Table 3 shows the FJS-12 score and gait parameters (Table 3).

### 3.2. Correlation Between the FJS-12 Score and Gait Parameters

Significant positive correlations were observed between the FJS-12 score and walking speed (r_s_ = 0.38, *p* < 0.05) as well as stride length (r_s_ = 0.51, *p* < 0.01). In addition, significant negative correlations were noted between the FJS-12 score and walking speed CV (r_s_ = −0.34, *p* < 0.05) as well as stride length CV (r_s_ = −0.35, *p* < 0.05).

A power analysis of the correlation between the FJS-12 score and each gait parameter was conducted (effect size: r, α error probability = 0.05, total sample size: 44). Among the gait parameters, stride length had the highest power (1-β) at 0.85. Table 4 shows the correlation between gait parameters and the FJS-12 score (Table 4).

### 3.3. Multiple Regression Analysis of the FJS-12 Score and Gait Parameters

In the correlation analysis between the FJS-12 score and gait parameters, all gait parameters were significantly correlated with the FJS-12 scores. Therefore, all gait parameters were included in a forced entry multiple regression analysis. In addition, age was included as a covariate to adjust for potential confounding effects. A stepwise multiple regression analysis was then conducted.

The results revealed that only stride length (β = 0.48, *p* < 0.01) was a significant predictor of the FJS-12 score among the independent variables. A power analysis of the multiple regression model (effect size R^2^ = 0.23, α error probability = 0.05, total sample size: 44) revealed a power (1-β) of 0.66. Table 5 shows the effects of gait parameters on the FJS-12 score based on a stepwise regression analysis.

### 3.4. ROC Curve

**Figure 2:**
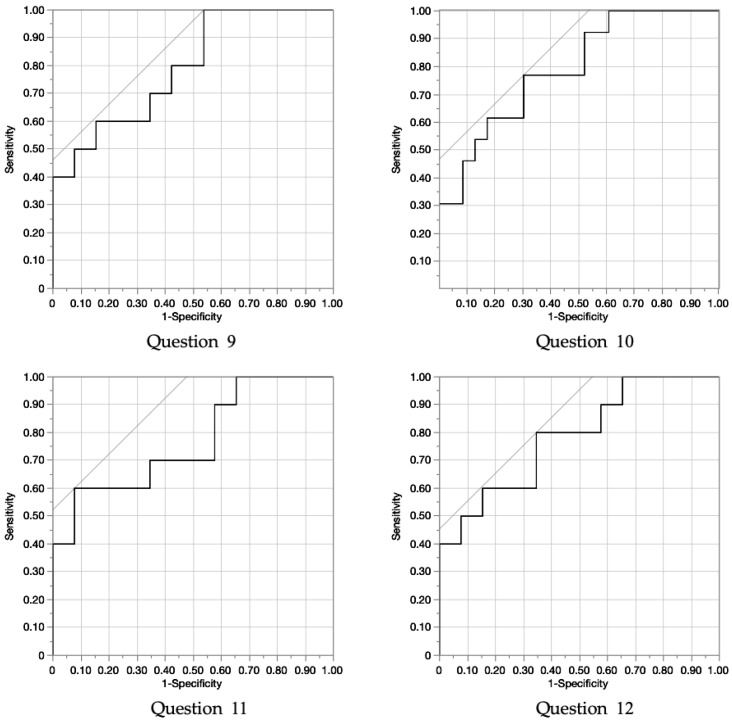
Receiver operator personality (ROC) analysis of the FJS-12 score and stride length (AUC ≥ 0.7). (Question 9) when you are standing for long periods of time? (Question 10) when you are doing the housework or gardening? (Question 11) when you are walking/hiking? (Question 12) when you are doing your favorite sport?

ROC curve analysis was performed to determine the cutoff stride length value for predicting discomfort using the FJS-12 subscales (Figure 2). The results revealed that the AUC was moderate (≥0.7) for the following FJS-12 items: Question 9—0.81; Question 10—0.75; Question 11—0.74; and Question 12—0.78. Table 6 shows the ROC curves of the FJS-12 score and stride length.

## 4. Discussion

The current study aimed to elucidate the relationship between the FJS-12 score and early postoperative gait parameters (temporal and distance) in patients who underwent THA. Further, the cutoff gait parameter values for predicting discomfort using the FJS subscales were examined. Multiple regression analysis revealed a significant association between the FJS-12 score and stride length. Moreover, ROC curve analysis showed that the accuracy of the cutoff stride length value was moderate or higher for the FJS-12 subscale items 9, 10, 11, and 12.

The FJS, developed based on the patient’s ability to forget their artificial joint, is used to evaluate postoperative outcomes after joint replacement surgery [24,25]. In this study, the mean FJS-12 score was 62.3 ± 29.7. Previous studies have reported FJS-12 scores of 68.6 ± 29.4 at 1.75 years after THA (*n* = 639, mean age: 63.5 ± 10.8 years, male patients: *n* = 339, female patients: *n* = 441) [8], and 63.8 ± 29.2 at 1–3 years after THA in another cohort (*n* = 329, mean age: 58.5 ± 9.8 years, male patients: 106, female patients: 223) [26]. Compared with the findings of these studies, ours were slightly lower. Based on previous research, the minimum cutoff values for a satisfactory health status (Patient Acceptable Symptom State [PASS]) ranged from 69.8 to 91.7 [27]. Galea et al. reported [28] that the PASS values for the FJS-12 score at 3 months and 1 and 2 years after THA were 59, 68, and 69 points, respectively, in a cohort of patients who underwent THA (*n* = 230, mean age: 68.0 ± 11 years, male participants: *n* = 61, female participants: *n* = 140). The FJS-12 score of patients who underwent THA at the 2-year postoperative period in our study might be slightly lower than that in previous studies and does not reach the PASS. These results might be influenced by factors such as age and sex. Giesinger et al. have shown that the FJS scores are more likely to decrease with age due to differences in joint perception [20] and recovery rates. In addition, male patients are generally more likely to have higher FJS-12 scores than female patients [29,30]. Our results might differ from those of previous studies who included younger and male participants.

Next, significant correlations were observed between the FJS-12 score and walking speed, stride length, walking speed CV, and stride length CV. In particular, the detection power of stride length was >0.8, which is higher than that of other parameters. Several reports have revealed significant improvements in comfortable walking speed up to approximately 1 year after THA [31,32,33,34]. Shibuya et al. reported [31] that postoperative walking speed in patients with THA is closely related to age, sex, muscle strength, and range of motion (ROM) of the hip and knee joints, with fast walkers exhibiting a significantly rapid functional recovery. Furthermore, consistently high recovery rates were observed even after adjusting for age, which is a confounding factor. Hence, walking speed should be used to predict functional recovery after THA [29]. Compared to preoperative measurements, stride length significantly increases during the stance phase of walking after THA [35], and ROM during hip extension continues to improve up to 12 months postoperatively [36]. Patients with HOA exhibit increased walking speed CV and stride length CV due to a reduced step length and stance phase duration on the affected side, decreased walking rate, increased trunk sway, and reduced maximum hip extension angle [36,37,38,39]. Hausdorff et al. [40] noted that gait variability in older people, reflected by step-to-step fluctuations, is strongly associated with muscle strength, balance, and walking speed. Therefore, walking speed, stride length, walking speed CV, and stride length CV can reflect the extent of physical function recovery after THA. The high detection power of stride length (>0.8) indicates a reliable correlation with the FJS-12 score [23].

Based on multiple regression analysis and adjustment for age, stride length emerged as the most influential factor for the FJS-12 score. Therefore, focusing on the FJS-12 subitems, the accuracy of the cutoff stride lengths was evaluated using ROC curves for the groups with and without discomfort. Among the 12 questions, the results showed moderate or higher AUC accuracy for questions 9–12. The specific content and cutoff values for these questions were as follows: are you aware of your artificial joint… (Question 9) when standing for a long period (83.0 cm), (Question 10) when doing housework or gardening (97.0 cm), (Question 11) when walking or hiking (78.2 cm), and (Question 12) when playing your favorite sport (97.0 cm). The cutoff values indicate that a stride length between 78.2 and 97.0 cm is needed to avoid being conscious of the artificial joint during the activities described in questions 9–12.

Previous studies have reported that the cutoff stride length value for predicting fall risk in community-dwelling elderly individuals is 64 cm [41]. By contrast, based on a study targeting patients with THA, the stride length at 3 months after surgery was 57.5 ± 5.9 cm [42]. The cutoff stride length values in this study were significantly higher than those in previous studies. This discrepancy might be attributed to the nature of the FJS-12 questions, which progressively require higher levels of physical activity from question 1 to question 12. In our study, questions 9–12, which showed significant results, corresponded to higher physical activity levels. Therefore, improving stride length could enhance satisfaction with daily activities requiring higher physical activity levels.

According to a systematic review focusing on the physical activity levels after THA, patients can have higher physical activity levels at 1 year postoperatively [43]. Further, improving physical activity levels after THA can enhance bone quality, improve the fixation of the artificial joint, and reduce the incidence of loosening [44]. Therefore, it is important to achieve higher levels of physical activity after surgery, and improving stride length is important to achieving higher physical activity levels.

This study had several limitations. First, it was a retrospective observational study conducted at a single institution with a small sample size (*n* = 44), which limits the generalizability of the results and statistical power, including the ability to compare findings with other populations. Additionally, a response rate of 14% for the 2-year FJS assessment indicates the potential for bias in the sample population, which may further affect the generalizability of the results. To address these limitations, larger, multicenter prospective studies must be conducted to enhance generalizability and statistical power. Increasing the follow-up response rate is also crucial to reduce bias. Moreover, future studies should perform more comprehensive adjustments of confounding factors to ensure more accurate results. Incorporating analyses of activities of daily living may provide an enhanced understanding of the relationship between gait parameters and the FJS-12 score. Second, this study did not analyze the impact of comorbidities assessed based on the Charlson Comorbidity Index, which may have influenced postoperative functional recovery. Future studies should explore the prevalence of comorbidities in detail. The present study examined the association between early postoperative gait ability and FJS-12 scores 2 years postoperatively, indicating that early rehabilitation is associated with improved walking ability. However, further research is warranted to clarify the causal relationship between these factors.

## 5. Conclusions

The improved walking ability of patients who underwent THA through early rehabilitation is associated with joint discomfort and patient satisfaction in daily life 2 years postoperatively.

## Figures and Tables

**Table 1:** Comparison of the baseline data of the participants (*n* = 44).

Age (years)	64.2	±	10.3
Height (cm)	153.4	±	7.4
Weight (kg)	57.5	±	15.9
BMI (kg/m^2^)	24.3	±	5.8
Affected side (left/right)	22	/	22

Mean ± standard deviation. BMI: body mass index.

**Table 2:** Forgotten Joint Score-12.

Are You Aware of Your Artificial Joint…
1. ...in bed at night?
2. ...when you are sitting on a chair for more than 1 h?
3. ...when you are walking for more than 15 min?
4. ...when you are taking a bath/shower?
5. ...when you are traveling in a car?
6. ...when you are climbing the stairs?
7. ...when you are walking on uneven ground?
8. ...when you are standing up from a low-sitting position?
9. ...when you are standing for long periods of time?
10. ...when you are doing the housework or gardening?
11. ...when you are walking/hiking?
12. ...when you are doing your favorite sport?

**Table 3:** FJS and gait parameter results.

Measures			
FJS	62.3	±	29.7
Gait speed (cm/s)	80.9	±	21.1
Stride length (cm)	96.5	±	17.6
Gait speed CV (%)	7.6	±	4.6
Stride length CV (%)	6.4	±	5.1

Continuous values are expressed as mean ± standard deviation. FJS: forgotten joint score, CV: coefficient of variation.

**Table 4:** Correlation between FJS and gait parameters.

	r_s_	*p* Value	Power (1-β)
Gait speed (cm/s)	0.38	0.02	0.73
Stride length (cm)	0.51	0.001	0.85
Gait speed CV (%)	−0.34	0.04	0.63
Stride length CV (%)	−0.35	0.03	0.62

CV: coefficient of variation.

**Table 5:** Results of stepwise regression analysis of gait parameters affecting FJS (*n* = 44).

Dependent Variables	Multiple Linear Regression				
	R^2^	β	*p* Value	VIF	Power (1-β)
Gait speed (cm/s)	0.27		0.27	6.11	0.74
Stride length (cm)	0.23	0.48	0.04	7.58	0.66
Gait speed CV (%)	0.30		0.69	1.75	0.79
Stride length CV (%)	0.30		0.89	2.48	0.79

CV: coefficient of variation; VIF: variance inflation factor.

**Table 6:** ROC curve of stride and FJS sub-items.

FJS Sub-Items	AUC	*p* Value	Cutoff Point	Sensitivity	Specificity
Question 9	0.81	0.007	83.0	0.63	0.48
Question 10	0.75	0.009	97.0	0.76	0.43
Question 11	0.74	0.002	78.2	0.6	0.48
Question 12	0.78	0.016	97.0	0.8	0.45

AUC: area under the curve; ROC: receiver operating characteristic. *p* < 0.05 according to ROC curve.

## Data Availability

Data are contained within the article.
